# Immunity raised by recent European subtype 1 PRRSV strains allows better replication of East European subtype 3 PRRSV strain Lena than that raised by an older strain

**DOI:** 10.1186/s13567-015-0292-y

**Published:** 2016-01-08

**Authors:** Ivan Trus, Ilias S. Frydas, Vishwanatha R. A. P. Reddy, Caroline Bonckaert, Yewei Li, Lise K. Kvisgaard, Lars E. Larsen, Hans J. Nauwynck

**Affiliations:** Laboratory of Virology, Faculty of Veterinary Medicine, Ghent University, Merelbeke, Belgium; National Veterinary Institute, Technical University of Denmark, Frederiksberg C, Denmark

## Abstract

Stable spatial distribution of porcine reproductive and respiratory syndrome (PRRSV)-1 subtypes in Europe is accompanied by a strong population immunity induced by local PRRSV strains. In the present study, it was examined if the immunity induced by three West European subtype 1 PRRSV strains (2007 isolate 07V063 and 2013 isolates 13V091 and 13V117) offers protection against the highly virulent East European subtype 3 PRRSV strain Lena. The number of fever days was greater (*p* < 0.05) in the control group (7.6 ± 1.7 days) compared to the immune groups (07V063-immune: 4.0 ± 1.2 days, 13V091-immune: 4.6 ± 1.1 days, 13V117-immune: 4.0 ± 2.9 days). In all groups, protection was characterized by reduction (*p* < 0.05) of AUC values of nasal shedding (control: 14.6, 07V063-immune: 3.4, 13V091-immune: 8.9, 13V117-immune: 8.0) and viremia (control: 28.1, 07V063-immune: 5.4, 13V091-immune: 9.0, 13V117-immune: 8.3). Reduction of respiratory disease, nasal shedding (mean AUC and mean peak values) and viremia (mean AUC and mean peak values) was more pronounced in 07V063-immune (*p* < 0.05) than in 13V091-immune and 13V117-immune animals. Inoculation with subtype 1 PRRSV strains caused priming of the Lena-specific virus neutralization antibody response. Upon challenge with Lena, we observed a very strong serological booster effect for neutralizing antibodies against strains used for the first inoculation. Our results indicate that inoculation with subtype 1 PRRSV strains can partially protect against antigenically divergent subtype 3 strains. The lower protection level elicited by recently isolated subtype 1 PRRSV strains may impair the outcome of the spatial expansion of subtype 3 strains from East Europe to West Europe.

## Introduction

Porcine reproductive and respiratory syndrome virus (PRRSV) is prevalent in most swine farms worldwide, and is a major cause of economic losses and animal suffering. Current genetic analysis of a number of European genotype PRRSV-1 strains reveals the existence of four different subtypes [1]. In Europe, a geographical demarcation exists between areas of low (Western and Central Europe) and high (Eastern Europe) PRRSV-1 diversity [1]. New Belgian PRRSV-1 variants still belong to subtype 1, but genetic changes have led to an increase in virulence and pathogenicity [2]. This drift has resulted in further economic losses in the swine industry in 2013 and 2014. Stability of the spatial distribution of different PRRSV subtypes in Europe allows us to continue using the term “East European subtypes” for subtypes 2, 3 and 4. However, there is a potential risk that East European subtypes, which are genetically and antigenically distinct from Pan-European subtype 1 viruses and are even more virulent/pathogenic, could emerge in Western and Central Europe, leading to a real catastrophe [1, 3, 4]. The role of existing herd immunity in keeping subtypes 2, 3 and 4 out of Western and Central Europe is of great interest.

Active immunization is currently the only widely available and approved way to control PRRS-related problems in swine herds worldwide. The existence of different PRRSV subtypes requires vaccines that induce a strong cross-protective immune response. In a recent publication, it was demonstrated that the PRRSV-1 subtype 1 vaccine strain modified live virus (MLV)-DV (GenBank: KJ127878), which is closely related to the old prototype virus LV (98.4% identity of full genome sequences) (GenBank: M96262), was able to give partial clinical and virologic protection against PRRSV-1 subtype 3 strain Lena [3]. With the appearance of genetically more distant PRRSV strains, the question raised as to what degree these strains induce a protective immunity against PRRSV Lena.

In the present study, the level of protection against European PRRSV subtype 3 strain Lena was examined in animals immunized with one old and two recent subtype 1 PRRSV-1 strains.

## Materials and methods

### Animals, experimental design and inoculation viruses

Twenty conventional pigs were obtained from a PRRS-negative farm. All animals were housed in separate stables in a biosafety level 2 (BSL2) facility at the Faculty of Veterinary Medicine, Ghent University, Belgium. No relevant pathogens (PRRSV, SIV, PCV2) were detected in the animals.

Pigs were randomly divided into four groups with five animals per group (07V063, 13V091, 13V117, and a mock-inoculated control group). Two consecutive inoculations were performed in this experiment. Pigs were 11 weeks old at the first inoculation and 18 weeks old at the second inoculation. Inoculations were performed intranasally using a 2 × 10^5^ tissue culture infectious dose (TCID) with a 50% end point (TCID_50_) PRRSV. The 07V063 group was inoculated with the third passage of 07V063 strain: the 13V091 group with the third passage of 13V091 strain; and the 13V117 group with the second passage of 13V117 strain; all propagated in porcine alveolar macrophages (PAMs). These subtype 1 PRRSV-1 strains were isolated from different Belgian farms in 2007 (07V063) and 2013 (13V091 and 13V117). Subtype 1 PRRSV 07V063 strain is a mildly pathogenic Belgian strain [2, 4]. Newly isolated strains originated from farms with animals experiencing endemic respiratory disorders in nursery pigs (13V091 and 13V117) [2]. The control group was inoculated intranasally using 1 mL phosphate-buffered saline (PBS) per nostril.

Seven weeks after the first inoculation all four groups were inoculated with the PRRSV Lena strain (forth passage propagated in PAMs). Lena is a highly pathogenic East European subtype 3 PRRSV strain isolated from aborted fetuses on a Belarusian farm [3–5]. Individual sterile syringes and plastic cannulas (Jorgenson Labs J12) were used for intranasal virus inoculation.

The sequences of 07V063, 13V091, 13V117 and Lena were downloaded from GenBank (GenBank: GU737264, KT159248, KT159249, JF802085). Alignment and phylogenetic analysis were performed using the Mobyle@Pasteur web bioinformatics framework [6].

### Clinical and pathological examinations

Clinical monitoring was performed on a daily basis from 3 days before challenge until 21 days post-challenge (dpc). Local parameters included respiratory disorders, discoloration of ears, presence or absence of periocular edema, and diarrhea. Systemic parameters included rectal temperature (with the threshold for fever set at 39.5 °C) and liveliness. Local parameters and liveliness were expressed as a score at the animal level, whereas diarrhea was evaluated on a group level. The respiratory scoring system was adapted from Karniychuk et al. [4].

After euthanasia at 35 dpc, lungs were collected and a numerical score was given based on the observed macroscopic lung lesions. This score was used to estimate the percentage of lungs affected by pneumonia, as previously described [7].

### Virus titration

Nasal secretions were collected at 0, 3, 5, 7, 10, 14, 21, 28 and 35 dpc using sterile swabs (COPAN 160C, Copan Italia SpA). One swab was used per nostril and per pig, and the two swabs were pooled. One mL of transport medium was added and after vortexing (1 h, 4 °C) the supernatant was collected and stored at −70 °C for virus titration. Transport medium was based on buffer solution [1× Dulbecco’s PBS (DPBS) with 0.9 mM CaCl_2_, 0.5 mM MgCl_2_ × 6H_2_O and 20 mg/L phenol red] supplemented with 10% fetal calf serum (FCS) and a mixture of antibiotics (1000 IU/mL penicillin, 1 mg/mL streptomycin and 0.5 mg/mL gentamycin). Virus titers in the supernatant and serum were determined by titration on PAMs collected from PRRSV negative pigs, as previously described [8]. The monoclonal antibody 13E2 against the nucleocapsid protein of PRRSV was used to detect PRRSV-infected cells. Plain swabs were weighed before and after swabbing, and viral titers were calculated per 100 mg of secrete [4]. Fifty percent endpoint titers were calculated by the Reed–Muench method [9].

### Serological examinations

Blood was collected at 0, 3, 5, 7, 10, 14, 21, 28 and 35 dpc by puncture of the *vena cava cranialis*, and serum samples were prepared after centrifugation (10 min, 1800 *g*, 4 °C). PRRSV-specific antibodies were detected by the immunoperoxidase monolayer assay (IPMA), and PRRSV-specific neutralizing antibodies were titrated using the virus neutralization (VN) test on MARC-145 cells, as previously described [10]. Serum samples were heat-inactivated for 30 min at 56 °C prior to testing. The PRRSV strain Lena was used as an antigen in IPMA and VN assays. Additional VN assays were performed with PRRSV strains 07V063, 13V091 and 13V117 to detect neutralizing antibodies in animals immunized with the respective strains.

### Statistical analysis

Serological titers (IPMA and VN), as well as viral loads, were log-transformed prior to analysis. Samples testing negative were assigned a value corresponding to half the minimum detectable titer. Gross pathology scores and area under the curve (AUC) were analyzed using the non-parametric Kruskal–Wallis test with Dunn’s post-test. Statistical analysis of continuous data was performed using repeated-measures two-way analysis of variance (rANOVA), with Bonferroni’s post-test. Results were considered to be significantly different at *p* < 0.05. All data were expressed as the mean value ± standard deviation (SD).

## Results

### Genetic relationship between subtype 1 strains and East European subtype 3 PRRSV strains

**Figure 1:**
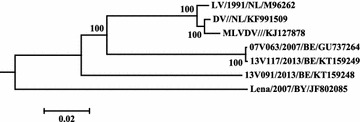
**A phylogenetic tree of circulating Belgian virus isolates.** Molecular phylogenetic analysis of full genome nucleotide sequences was constructed using the Neighbor-Joining method. Phylogenetic relationships were estimated using the Clustal Omega method. Prototype subtype 1 (LV) and subtype 3 (Lena) PRRSV-1 strains, vaccine parental (DV) and attenuated (MLV-DV) PRRSV-1 strains were added to phylogenetic tree. The optimal tree is drawn to scale. Numbers indicate bootstrap values of 100 replicates. Strain nomenclature is as follows: name or diagnostic number/year of isolation/country of origin/GenBank accesion number.

A low level of identity was calculated between the pairwise distances of full genome sequences of the PRRSV subtype 3 Lena strain and the PRRSV subtype 1 strains 07V063 (79.9%), 13V091 (79.6%) and 13V117 (79.8%) (Figure 1).

Phylogenetic analysis of full genome sequences revealed a high identity level (99.9%) between the 07V063 and 13V117 strains (Figure 1). Non-synonymous nucleotide substitutions resulted in eight different amino acids located in non-structural protein (nsp) 1 (V140M), nsp2 (H786L, G1300S), nsp7 (S2192N), nsp9 (M72I, M266V), GP3 (N253H) and GP4 (Q72P). Two were located in putative antigenic epitopes [11–13]. Q72P was found in the B cell ES12 epitope and neutralizing antigenic region (NAR) GP4.16 [11, 12]. N253H was found in NAR GP3.62 [12].

### Clinical signs and pathological examination

**Figure 2:**
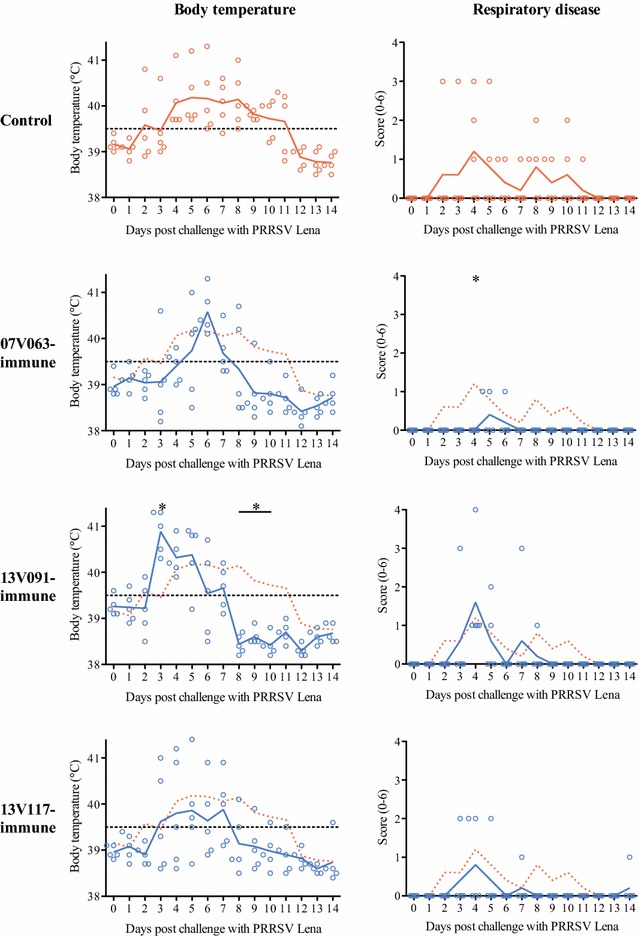
**Body temperature and respiratory disease scoring after challenge.** Lines represent the mean value in each group. Dotted line gives the mean values of the control group. Temperatures of ≥39.5 °C were considered as fever (dashed line). Respiratory disease scores ranged from 0 to 6: 0 = normal; 1 = mild dyspnea and/or tachypnea when stressed; 2 = mild dyspnea and/or tachypnea at rest; 3 = moderate dyspnea and/or tachypnea when stressed; 4 = moderate dyspnea and/or tachypnea at rest; 5 = severe dyspnea and/or tachypnea when stressed; 6 = severe dyspnea and/or tachypnea at rest. Asterisk represents statistically discernible differences from the control (*p* < 0.05).

Body temperature and respiratory disease scores are represented in Figure 2. Fever was observed after challenge with PRRSV Lena in animals from 2 to 11 dpc in the control group, from 3 to 9 dpc in the 07V063-immune group, from 3 to 7 dpc in the 13V091-immune group, and from 3 to 8 dpc in the 13V117-immune group. The number of fever days was higher (*p* < 0.05) in the control group (7.6 ± 1.7 days) compared to the immune groups (07V063-immune: 4.0 ± 1.2 days, 13V091-immune: 4.6 ± 1.1 days, 13V117-immune: 4.0 ± 2.9 days). Significantly higher mean temperature (*p* < 0.01) was observed in the 13V091-immune group (40.9 ± 0.5 °C) compared to the control group (39.5 ± 0.7 °C) at 3 dpc. Mean AUC values had no significant differences between the groups (control group: 4.5 ± 2.9, 07V063-immune group: 2.4 ± 0.9, 13V091-immune group: 3.6 ± 1.2, 13V117-immune group: 2.5 ± 2.6). In all groups, animals showed no diarrhea, nasal discharge, coughing, or ear discoloration.

Animals from the 07V063-immune group showed significantly lower mean clinical scores for respiratory disease than control animals at 3 dpc (*p* < 0.05). One animal from the 13V091-immune group, and three animals from the control and 13V117-immune groups had clinical scores >1. No animals from the 07V063-immune group had such a level of respiratory disease.

The extent of macroscopic pneumonia was not significantly different (*p* > 0.05). The lowest values were observed in the 07V063-immune group (control group: 1.6 ± 1.8%, 07V063-immune group: 0.3 ± 0.4%, 13V091-immune group: 1.0 ± 2.2%, 13V117-immune group: 1.5 ± 1.4%).

### Virological analysis

**Figure 3:**
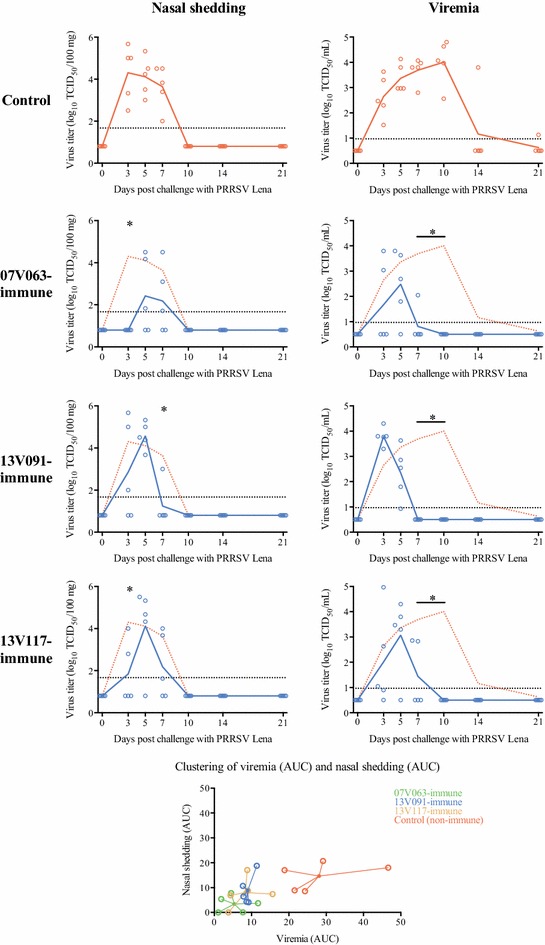
**PRRSV titers in nasal secretions and sera after challenge and clustering of individual AUCs.** Lines represent the mean titer in each group. Dotted line gives the mean values for the control group. Dashed line gives the detection limit for virus titration. Asterisk represents statistically discernible differences from the control (*p* < 0.05).

Nasal shedding (Figure 3) was observed from 3 dpc in the control, 13V091-immune and 13V117-immune animals, and from 5 dpc in animals from the 07V063-immune group. At 5–7 dpc, shedding was found in all animals except for one pig from the 13V117-immune group. A peak mean virus titer was reached at 3 (control group: 10^4.3^ TCID_50_/100 mg) or 5 dpc (07V063-immune group: 10^2.4^ TCID_50_/100 mg, 13V091-immune group: 10^4.6^ TCID_50_/100 mg, 13V117-immune group: 10^4.1^ TCID_50_/100 mg). Significantly lower mean virus titers were detected at 3 dpc in 07V063-immune and 13V117-immune animals, and at 7 dpc in 13V091-immune animals compared to control pigs. Nasal shedding stopped in all animals by 10 dpc. The mean AUC was significantly lower (*p* < 0.05) in the 07V063-immune group than in the control group (control group: 14.6 ± 5.6, 07V063-immune group: 3.4 ± 3.4, 13V091-immune group: 8.9 ± 6.1, 13V117-immune group: 8.0 ± 6.1) (Figure 3).

Viremia was detected from 3 dpc in all animals, except for one 13V117-immune animal and in three animals from the 07V063-immune group (Figure 3). Virus was isolated from all animals at 5–7 dpc. A peak mean virus titer was observed at 10 dpc (control group: 10^4.0^ TCID_50_/mL), 5 dpc (07V063-immune group: 10^2.5^ TCID_50_/mL, 13V117-immune group: 10^3.1^ TCID_50_/mL) or 3 dpc (13V091-immune group: 10^3.8^ TCID_50_/mL). Significantly higher virus titers were found in the control group compared to all immune groups at 7 and 10 dpc (*p* < 0.001). No virus was isolated from serum samples starting from 28 dpc (control group), 10 dpc (07V063-immune and 13V117-immune groups) or 7 dpc (13V091-immune group). The mean AUC was significantly higher (*p* < 0.05) in control animals than in 07V063-immune and 13V117-immune groups (control group: 28.1 ± 11.0, 07V063-immune group: 5.4 ± 4.4, 13V091-immune group: 9.0 ± 1.5, 13V117-immune group: 8.3 ± 4.8). The relationship between the AUCs of nasal shedding and viremia was found to be significant (Spearman’s rho = 0.62, *p* = 0.0038) (Figure 3).

### Serology

**Figure 4:**
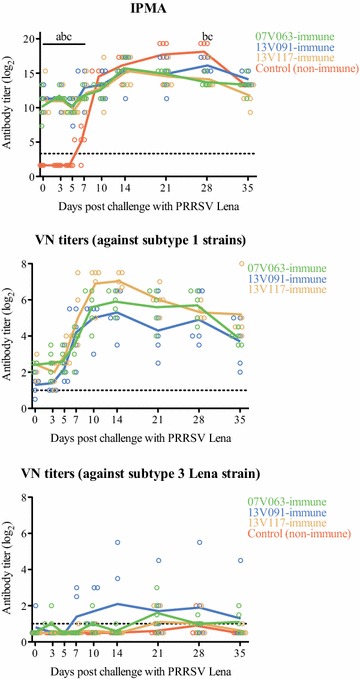
**PRRSV-specific IPMA and VN antibody titers after challenge.** The PRRSV Lena strain was used as an antigen in IPMA assay. VN antibody titers were tested against subtype 1 (07V063, 13V091 and 13V117, respectively) and against subtype 3 PRRSV (Lena) strains. Lines represent the mean titer in each group. Dashed line gives detection limit for the test. Letters (a: 13V091-immune group, b: 13V117-immune group, c: 07V063-immune group) represent statistically significant differences from the control (*p* < 0.05).

Prior to challenge, serum samples animals in the control group were negative according to the IPMA (Figure 4). Animals from all immune groups had high titers of virus-specific antibodies (2^7.3^–2^15.3^). The antibody titers in immune and control animals were not different from 10 dpc (*p* > 0.05).

VN antibodies against subtype 1 PRRSV strains (07V063, 13V091 and 13V117) were detected at 0 dpc in five animals from the 07V063-immune group, four animals from the 13V091-immune group and five out of five animals from the 13V117-immune group (Figure 4). Individual VN antibody titers increased fourfold at 7 dpc in 07V063, 13V091 and 13V117-immune groups.

Prior to challenge, VN antibodies against the subtype 3 PRRSV Lena strain were detected only in one animal from the 13V091-immune group (Figure 4). Upon challenge, two out of five animals in the control group had a detectable level of VN antibodies at 21 and 28 dpc. In immune groups, the majority of animals (>50%) demonstrated a VN antibody response against Lena strain during the experiment: 07V063-immune group: five animals, 13V091-immune group: four animals, 13V117-immune group: three animals).

## Discussion

A high rate of genetic and antigenic variability among PRRSV isolates hampers effective prevention and control of the disease by the use of commercial vaccines. Rising diversity of PRRSV makes it impossible to have a reference isolate representing the whole country or a part of Europe. In the present study, we examined the protection of the immunity induced by an old and two new West European subtype 1 PRRSV strains effective against a heterologous East European subtype 3 virus challenge. The three subtype 1 isolates that were used in this study (07V063, 13V091 and 13V117) differed strongly genetically from subtype 3 PRRSV strain Lena. Detailed reports on their genetic backgrounds and the pathogenicity of these subtype 1 and 3 PRRSV strains have been previously published [1–5].

As an outcome of this study, protection in all immunized groups was characterized by reduction of fever length (*p* < 0.05), nasal shedding (mean AUC) and viremia (mean AUC). Reduction of respiratory disease, nasal shedding (mean AUC and mean peak values) and viremia (mean AUC and mean peak values) was more pronounced (*p* < 0.05) in animals inoculated with the older, low pathogenic strain (07V063-immune group). Subtype 1 PRRSV 13V091 and 13V117 strains were isolated in 2013, 6 years later than the 07V063 strain. A lower level of cross protection caused by immunization with the most recent strains, illustrates a negative impact of rising evolutional diversity of contemporary PRRSV strains on the formation of heterosubtypic immunological protection in swine herds, and on the preservation of spatial distribution of PRRSV subtypes in Europe.

Cellular immunity and neutralizing antibodies may be involved in clearance of the virus after PRRSV infection. The latter may correlate with the protective immunity against PRRSV [14]. In this study, inoculation of animals with subtype 1 PRRSV strains caused priming of Lena-specific VN antibodies. We observed a faster VN antibody response upon challenge with PRRSV Lena in the 07V063-immune group (50% of the animals formed a VN antibody response at 3 dpc) compared to the other groups (control group: >35 dpc, 13V091-immune group: 10 dpc, 13V117-immune group: 21 dpc). Moreover, only in the 07V063-immune group was a VN antibody response against PRRSV Lena detected in all animals. Faster VN antibody response in animals from the 07V063-immune group coincides with better clinical and virologic protection. Thus, VN antibody response upon challenge may be considered as an important factor in the development of immunologic protection.

Upon challenge with subtype 3 PRRSV Lena, a booster effect was observed against strains used for the first inoculation. Analysis of putative antigen recognition domains revealed conservation for two of them [11–13]. One neutralizing antigenic region (GP2.30) was conserved in all of the strains used in this study (EHSGQAAWKQVV) [12]. One epitope (HGAGNMGVDGSVWDF) from nsp9 with T-cell antigen reactivity was conserved in Lena, 07V063 and 13V117 strains [13]. Although subtype 3 strain Lena is genetically and antigenically quite different from the subtype 1 PRRSV strains (07V063, 13V091 and 13V117), neutralizing epitopes (e.g., GP2.30) may stay conserved and provide the VN antibody booster reaction.

Possible exacerbation of clinical effects [15, 16], intensive interstitial pneumonia [17] and increased viremia [18] has already been reported in animals immunized with DNA, sub-unit, and MLV vaccines against PRRS, respectively. We observed that immunization with the 07V063 strain, compared to the 13V091 and 13V117 strains, provided substantially better cross-protection against the subtype 3 PRRSV Lena strain. In contrast, animals from the 13V091-immune group demonstrated temporal exacerbation of PRRS disease manifested by higher body temperature (*p* < 0.001) and viremia (*p* < 0.05) at 3 dpc. Compared to the 07V063-immune group, 13V091-immune animals had a higher body temperature (*p* < 0.001), respiratory disease and viremia (*p* < 0.01) at 3 dpc, and higher virus titers in nasal secretions at 3–5 dpc (*p* < 0.05). Taken together, these data suggest that the immune response against PRRSV might be a double-edged sword [19]. On the one hand, it provides protection against genetically close and distant strains, but on the other hand it may activate early replication after contact by an antibody-dependent enhancement of infectivity (ADEI) process [18]. In the current study, genetic relatedness may not be a useful guide to accurately predict the level of cross-protection in pigs. Further research is needed to assess the mechanism involved in the immunopathogenesis of PRRS.

In the present study, immunization of animals with subtype 1 PRRSV strains resulted in partial protection, and significantly reduced viral replication and clinical signs upon inoculation with a heterologous East European subtype 3 PRRSV Lena strain. These results could be compared with the outcome of our recent study on the efficacy of an attenuated vaccine (Porcilis^®^ PRRS, MSD Animal Health) based on the subtype 1 PRRSV DV strain (GenBank: KJ127878, KF991509) [3]. PRRSV-specific IPMA antibody titers after one shot vaccination were similar to those observed at 0 dpc in the current study. Animals were challenged with subtype 3 PRRSV Lena strain in the same way as in this study. Protection in the vaccination study was characterized by a similar reduction of clinical disease, i.e., reduced fever length (*p* < 0.05) and respiratory distress level (*p* < 0.05). Virologic parameters showed protection with reduced viremia (*p* < 0.05) and shorter duration of nasal shedding (*p* < 0.05), and lower AUCs for viremia (*p* < 0.05) and nasal shedding (*p* < 0.05) as in the present study. Similarly to the current experiment, the majority (>50%) of vaccinated animals had detectable levels of VN antibodies after challenge (14 dpc for the vaccination at 4 weeks, 28 dpc for the vaccination at 7 weeks). Overall, we can conclude that parenteral administration of the widely used MLV vaccine against PRRS and immunization with subtype 1 strains is able to induce a comparable protection level against the subtype 3 PRRSV strain Lena.

Immunization of pigs should be used to prevent the transmission of recently isolated highly pathogenic PRRSV strains. However, currently available commercial vaccines based on old type 1 PRRSV strains provide only partial protection against genetically different strains from the same type [3, 20]. Therefore, the use of live-resident virus inoculation (LVI) may be proposed. This procedure, which has been evaluated as PRRS management tool [19, 21], consists of exposure of naïve gilts prior to introducing them into the breeding herd. Although the immune response to virulent PRRSV is usually stronger than that to attenuated virus [19], the LVI approach can increase the risk of further dissemination of field PRRSV strains. In addition, higher production losses in breeding herds were shown when the LVI method was used compared to farms using MLV vaccines [19]. Therefore, the use of MLV vaccines should be preferred for PRRS control and elimination programs.

The restricted flow of marketable pigs from Eastern to Western Europe is keeping the risk of transboundary spread of highly pathogenic subtype 3 PRRSV strains to a minimum. In our study we investigated the role of immunity on the outcome of such spread. Although the immunity elicited by all three subtype 1 PRRSV strains provides a partial protection against antigenically divergent subtype 3 strains, a lower protection level was induced by the two most recently isolated subtype 1 PRRSV strains (2013) compared to that induced by the older strain (2007). The higher replication of a subtype 3 PRRSV strain in pigs immune after infection with more recent strains might result in an easier spatial expansion of subtype 3 strains nowadays than in the past.

## Abbreviations

ADEI: antibody-dependent enhancement of infectivity
AUC: area under the curve
BSL2: biosafety level 2
DPBS: Dubecco’s phosphate buffered saline
dpc: days post-challenge
FCS: fetal calf serum
IPMA: immunoperoxidase monolayer assay
LVI: live-resident virus inoculation
MLV: modified live virus
nsp: non-structural protein
PAM: porcine alveolar macrophage
PBS: phosphate buffered saline
PRRS: porcine reproductive and respiratory syndrome
PRRSV: porcine reproductive and respiratory syndrome virus
rANOVA: repeated-measures two-way analysis of variance
SD: standard deviation
TCID: tissue culture infectious dose
VN: virus neutralization
